# Diet and sociodemographic predictors of the double burden of malnutrition in urban Zimbabwe

**DOI:** 10.4102/phcfm.v17i1.4834

**Published:** 2025-03-25

**Authors:** Simbarashe Kasanzu, Joconiah Chirenda, Anesu Marume

**Affiliations:** 1 Department of Global Public Health and Family Medicine, Faculty of Medicine and Health Sciences, University of Zimbabwe, Harare, Zimbabwe

**Keywords:** BMI, dietary patterns, food security, urban Zimbabwe, undernutrition, overnutrition

## Abstract

**Background:**

Rapid urbanisation in low- and middle-income countries (LMICs) has intensified the double burden of malnutrition, where undernutrition and overnutrition coexist in the same population

**Aim:**

This study aimed to examine the point prevalence rate and risk factors of the double burden of malnutrition among adults in urban Zimbabwe.

**Setting:**

The study was conducted in Zimbabwe’s two metropolitan provinces (Harare and Bulawayo).

**Methods:**

A cross-sectional study of 348 urban adults explored associations between dietary intake, socio-demographics and anthropometrics using means, frequencies, and logistic regression.

**Results:**

Obesity prevalence was 23.6%, and underweight prevalence was 8.6%. Men had higher odds of being underweight than women (Adjusted Odds Ratio 2.30, 95% CI 1.20–4.41), while high household income increased the odds of obesity (Adjusted Odds Ratio 2.90, 95% CI 1.47–5.60). A fruit and vegetable-rich diet reduced the odds of obesity by 47% (Adjusted Odds Ratio 0.53, 95% CI 0.26–0.89), whereas a diet dominated by staples and sugary foods increased the risk of obesity.

**Conclusion:**

Obesity and underweight were common among urban adults in Zimbabwe, where both undernutrition and overnutrition pose significant health risks. Public health interventions in LMICs should broaden their focus to address adult malnutrition and its contribution to diet-related non-communicable diseases (NCDs).

**Contribution:**

The double burden of malnutrition underscores an urgent need for comprehensive public health strategies in LMICs. Efforts should move beyond childhood undernutrition to address the entire spectrum of malnutrition. Tackling these challenges holistically will be key to mitigating undernutrition, curbing rising obesity rates, and, in turn, reversing the tide of diet-related NCDs.

## Introduction

The coexistence of undernutrition and overnutrition in the same communities presents a complex challenge for malnutrition prevention, management and control worldwide. This dual burden is particularly pronounced in low- and middle-income countries (LMICs), where the historical struggle with undernutrition now intersects with rising rates of obesity and diet-related non-communicable diseases (NCDs).^1^ The World Health Organization (WHO) reports that in 2022, an estimated 2.5 billion adults were overweight, with 890 million classified as obese, while 390m were underweight.^2^

Traditionally, malnutrition in LMICs has been characterised by undernutrition, particularly in rural areas where subsistence farming predominates.^3^ The reliance on farming output resulted in insufficient dietary diversity, with essential dietary components lacking within the diet and primarily fruit and village farm produce consumption.^3,4^ This predisposed rural communities to undernutrition and micronutrient deficiency. The urban communities had a measured level of protection as they had access to dietary options from the rural farming community and a wider variety of food options within supermarkets. However, globalisation, shifts in food availability and technological advances have driven a significant ‘nutrition transition’ in LMICS.^5,6^ The impact of the transition is most prominent in urban populations.^7^

The nutrition transition is defined as a shift from traditional diets, which were energy-dense and high in fibre, to more processed foods high in sugars and fats. This shift is influenced by globalisation, economic growth, urbanisation and changing social norms regarding food consumption.^8,9^ Aggressive marketing of processed and imported food products and a global preference for ‘modern’ dietary options have redefined consumption patterns and relegated traditional diets to a status perceived as inferior.^10,11^

Advances in technology have also improved the production of food items and, more often, focused on food items with global appeal, which has increased prices of food items produced locally and, in turn, has made available cheaper and sometimes less healthy options.^12^ The cost of traditional dietary options has made them less desirable. At the same time, the fact that they are not available in most retail outlets has reduced access to these options for people who rely primarily on store-bought dietary consumption instead of those practicing subsistence farming in rural areas.^13^ The available dietary options are quite energy-dense and have resulted in overnutrition, with a rising prevalence of overweight, obesity and diet-related NCDs.^14^

In addition to the nutrition transition, rapid urbanisation in LMICs has significantly reshaped food environments, dietary behaviours and lifestyle patterns.^5^ The conversion of arable land into residential and commercial spaces has limited the capacity for urban families to engage in small-scale farming, a key source of diversity in nutrition.^15^ Moreover, the economic constraints prevalent in many urban households in LMICs exacerbate these issues, as limited employment opportunities lead to food insecurity and poor dietary choices.

Despite these urgent needs, urban malnutrition has received limited attention in research and policy. Historically, most interventions and literature have focused on rural communities where social services are often under-resourced.^7,16,17^ This rural-centric approach has resulted in significant knowledge gaps in understanding the nuances of urban malnutrition in LMICs, including dietary sources and nutritional disparities within urban populations. While global studies on urban malnutrition exist, they often reflect the situation in higher-income settings, leaving a critical gap in data specific to LMICs.^18,19^

There are still gaps in evidence of nutrition transition, dietary options and the effect of diet on the double burden of malnutrition in LMICS. Urban areas represent a unique context, with extreme socio-economic diversity spanning affluent suburbs to impoverished slums. In light of the nutrition transition, urbanisation and socioeconomic diversity in LMIC urban settings, this study aims to investigate the dietary patterns and socio-economic determinants of malnutrition in urban Zimbabwe. This research will contribute to a better understanding of urban dietary trends and their impact on the dual burden of malnutrition, informing future interventions and policy development to address these complex challenges.

## Research methods and design

### Study design

A cross-sectional study was conducted in the two metropolitan provinces of Zimbabwe: Harare and Bulawayo.

### Study setting

Harare, the capital city of Zimbabwe, has a population of 2 487 209, while Bulawayo, the second-largest city, has a population of 1 665 995. Harare is predominantly Shona-speaking, whereas Bulawayo is a multi-ethnic province with residents speaking Ndebele, Shona, Tswana, Sotho and Kalanga. These provinces were selected as study sites because of their high population densities, urban settings and contrasting demographic profiles, providing an ideal context for exploring the double burden of malnutrition. Harare is a densely populated and economically vibrant metropolitan area, while Bulawayo has a lower population density and a more diverse ethnic composition, representing different socio-economic and cultural dynamics.

### Study population

The target population was any adult who had resided in either Harare or Bulawayo provinces for the past 6 months before data collection.

### Sampling strategy

The Dobson formula was used to estimate the study sample at 95% confidence, considering a national prevalence of stunting of 30% used as a proxy because of a lack of studies measuring adult malnutrition. The sample size was calculated to be 323 individuals. After adding a 10% provision for non-response, we interviewed 348 individuals. Participants were selected using a two-stage sampling process. In the first stage, relevant wards within each metropolitan province were identified. A list of wards for each province was compiled, and 10 wards were randomly selected using computer-generated numbers. In the second stage, systematic random sampling was used to identify households within the selected wards. A central point in each ward was established, and a random direction was chosen using a compass. Enumerators visited every nth household along this direction, with ‘*n*’ determined by dividing the total number of households by the desired sample size for each ward. The next household was approached if no adult was available at a selected household. This method ensured a representative population sample while remaining practical for data collection.

### Study variables

The dependent variables for the study were nutritional status (underweight, normal weight and obese) and dietary quality and quantity. Nutritional status was assessed using Body Mass Index (BMI), calculated as weight (kg) divided by height (m^2^). According to WHO guidelines, underweight was defined as a BMI < 18.5, normal weight as a BMI of 18.5–24.9, pre-obesity as a BMI of 25–29.9 and obesity as a BMI ≥ 30.^20^ The pre-obese and normal weight categories were combined into a single group for analysis. The independent variables for the study included sociodemographic factors (age, gender, education level, income), household characteristics (number of members, residency status) and lifestyle factors (physical activity, smoking status).

Household income status was assessed using principal component analysis (PCA): a statistical technique that reduces a large set of variables into principal components to identify patterns and summarise variability. It is widely used for deriving wealth indices in demographic and health studies.^21,22^ Respondents were asked about ownership of assets and amenities, including vehicles, household appliances and access to basic services. PCA was used to assign weights to these items, generating a composite wealth index that classified households into five income groups. For analysis, these groups were collapsed into three categories: low income (quintiles 1 and 2), middle income (quintile 3) and high income (quintiles 4 and 5).

Dietary intake patterns were assessed using PCA. Respondents completed a 24 h dietary recall and a food frequency questionnaire (FFQ), which captured the frequency of food item consumption over the past week and month. The FFQ questions were adapted from the Zimbabwe Livelihood Assessment tool, which had been validated for local settings using the Food and Agriculture Organizations’ (FAO) guidelines for dietary assessment.^23,24^ Principal component analysis was then employed to assign weights to the food items, generating composite dietary patterns that reflected distinct eating behaviours in the population.

### Data collection

Data were collected between April and August 2024 through in-person visits to the selected households. Trained research assistants conducted interviews and took anthropometric measurements. Structured questionnaires were administered to gather comprehensive sociodemographic and lifestyle information. Additionally, a validated FFQ was utilised to assess dietary patterns. Calibrated weighing scales and stadiometers were employed to ensure accurate anthropometric measurements.

Several quality control measures were implemented to maintain the integrity and reliability of the data. Research assistants underwent thorough training on standardised data-collection procedures to ensure consistency and accuracy in data collection. Measurement tools, such as weighing scales and stadiometers, were regularly calibrated to maintain precision. All data were cross-checked for discrepancies with constant supervisory checks to data-collection teams.

### Data management and analysis

Collected data were entered into a secure, password-protected database through Kobo Collect. Data cleaning was performed to identify and correct any inconsistencies or errors. Data analysis was conducted using R studio. The study encompassed several statistical methods to examine the data comprehensively. Descriptive statistics, including means, standard deviations, frequencies and proportions, were calculated to summarise the data and provide an overall picture of the study population. Inferential statistics were employed to assess associations between malnutrition and various socio-demographic factors. Following that, logistic regression analysis was performed to determine the relationship between diet and malnutrition, with socio-demographic factors identified as significant predictors of malnutrition controlled for confounding.

### Ethical considerations

Permission to conduct the study was obtained from the Ministry of Local Government through the Directors of Health Services for both Bulawayo City Council and Harare City Council. Ethical clearance to conduct this study was obtained from the the University of Zimbabwe Joint Research Ethics Committee on 22 May 2024. The ethical clearance number is JREC 326/2024. Written informed consent was obtained from all participants. Participants were told that their participation was voluntary and that they could withdraw without consequences. Participants were assured of the confidentiality of their identities. All data were anonymised, and personal identifiers were removed before analysis. Data were stored securely, and restricted access was provided only to the principal investigator.

## Results

A total of 348 respondents were interviewed, most of whom were women (52.9%). Nearly a quarter (23.6%) of the respondents were identified as obese, while 8.6% were identified as underweight. Most participants (63.5%) were below 35 years old, and 64.5% were from households with between 3 and 5 people. Household socioeconomic status showed varied distribution, with 43.7% from low-income, 34.2% from middle-income and 22.1% from high-income households. Most (75.0%) stated they had reached the high school level, and 77.0% were informally employed (Table 1).

**TABLE 1:** Socio-demographic characteristics of study participants.

Variables	Normal		Underweight		Obese	
	*n*	%	*n*	%	*n*	%
**Sex**						
Male	112	68.3	19	11.6	33	20.1
Female	124	67.4	11	6.0	49	26.6
**Age (years)**						
18–25	65	63.1	11	10.7	27	26.2
26–35	77	77.8	6	6.1	16	16.2
36–45	38	52.8	5	6.9	29	40.3
45 >>	56	75.7	8	10.8	10	13.5
**Number in household**						
< 3	49	61.3	6	7.5	25	31.2
3–5	89	64.5	11	8.0	38	27.5
> 5	98	75.4	13	10.0	19	14.6
**Location of residence**						
High density	109	62.3	14	8.0	52	29.7
Low-medium density	127	73.4	16	9.2	30	17.3
**Family wealth status**						
Low income	78	56.9	16	11.7	43	31.4
Middle income	68	69.4	9	9.2	21	21.4
High income	90	79.6	5	4.4	18	15.9
**Last education level**						
Less than high school	51	65.4	10	12.8	17	21.8
High school	102	75.0	7	5.1	27	19.9
Tertiary	83	61.9	13	9.7	38	28.4
**Employment status**						
Unemployed	61	65.6	13	14.0	19	20.4
Informal employment	114	77.0	6	4.1	28	18.9
Formal employment	61	57.0	11	10.3	35	32.7

### Socio-demographic predictors of the double burden of malnutrition

Men had 2.3 times the odds of being underweight than women (AOR 2.30, 95% CI 1.20–4.41). Although men had increased odds of obesity (AOR 1.80, 95% CI 0.72–4.48), this finding was not statistically significant (*p* = 0.24). Compared to the youngest age group (18–25 years), individuals aged 26–35 had significantly lower odds of being underweight (AOR 0.30, 95% CI 0.15–0.16). The odds of obesity were notably lower for individuals aged 45 and above (AOR 0.44, 95% CI 0.23–0.83).

Participants from households of three to five members were 1.43 times more likely to be obese (*p* < 0.001) compared to those from smaller households. Residing in low-medium density areas increased the odds of being underweight (AOR 1.33, 95% CI 1.14–3.61) but did not significantly affect obesity (AOR 0.68, 95% CI 0.30–1.55). Middle-income individuals were more likely to be underweight than those with low income (AOR 3.16, 95% CI 1.56–6.43), while high-income individuals had significantly greater odds of obesity (AOR 2.90, 95% CI 1.47–5.60). Participants reporting education attainment less than high school education had 24% reduced odds of being underweight (*p* = 0.27) compared to those with tertiary education. Unemployed participants had increased odds of obesity (AOR 1.81, 95% CI 1.24–3.63). Informal employment slightly increased the odds of being underweight (AOR 1.20, 95% CI 1.13–2.73, *p* = 0.05) but did not significantly impact obesity (AOR 0.94, 95% CI 0.34–2.55, *p* = 0.52) (Table 2).

**TABLE 2:** Factors associated with the double burden of malnutrition in urban Zimbabwe.

Variable	Underweight (BMI < 18.5)			Obese (BMI >30)		
	AOR	95%CI	*p*	AOR	95%CI	*p*
**Sex**						
Female	R	-	-	R	-	-
Male	2.30	1.20–4.41	0.03	1.80	0.72–4.48	0.24
**Age (years)**						
18–25	R	-	-	R	-	-
26–35	0.30	0.15–0.16	< 0.001	1.96	0.82–4.65	0.35
36–45	0.79	0.17–0.99	< 0.001	0.58	0.32–1.00	0.15
45 >>	0.61	0.26–1.42	0.19	0.44	0.23–0.83	0.02
**Number in household**						
< 3	R	-	-	R	-	-
3–5	1.35	0.48–3.12	0.09	1.43	1.27–3.59	< 0.001
> 5	0.84	0.29–2.40	0.23	1.14	0.33–4.23	0.15
**Location of residence**						
High density	R	-	-	R	-	-
Low-medium density	1.33	1.14–3.61	0.02	0.68	0.30–1.55	0.10
**Family wealth status**						
Low income	R	-	-	R	-	-
Middle income	3.16	1.56–6.43	0.05	0.55	0.10–3.16	0.25
High income	1.39	0.37–5.18	0.15	2.90	1.47–5.60	< 0.001
**Last education level**						
Less than high school	0.76	0.40–1.44	0.27	0.35	0.18–0.67	0.01
High school	1.32	0.70–2.51	0.34	2.49	0.27–4.88	0.34
Tertiary	R	-	-	R	-	-
**Employment status**						
Unemployed	0.71	0.89–2.47	0.08	1.81	1.24–3.63	< 0.001
Informal employment	1.20	1.13–2.73	0.05	0.94	0.34–2.55	0.52
Formal employment	R	-	-	-	-	-

BMI, body mass index; AOR, adjusted odds ratio; Cl, confidence interval.

### Diet and the double burden of malnutrition

Food items considered under the classification of Starch, Bread and Cereals had the highest frequency of intake, with 78% highlighting daily consumption. Milk and milk products were the second most frequently consumed category, followed by meat and poultry. While not consumed as daily components, they are regular items in the overall diet. Vegetables and fruits were generally consumed with moderate frequency. Weekly consumption of these groups (1–3 times per week) was common, although less frequent daily intake was observed compared to starches and milk products.

**FIGURE 1:**
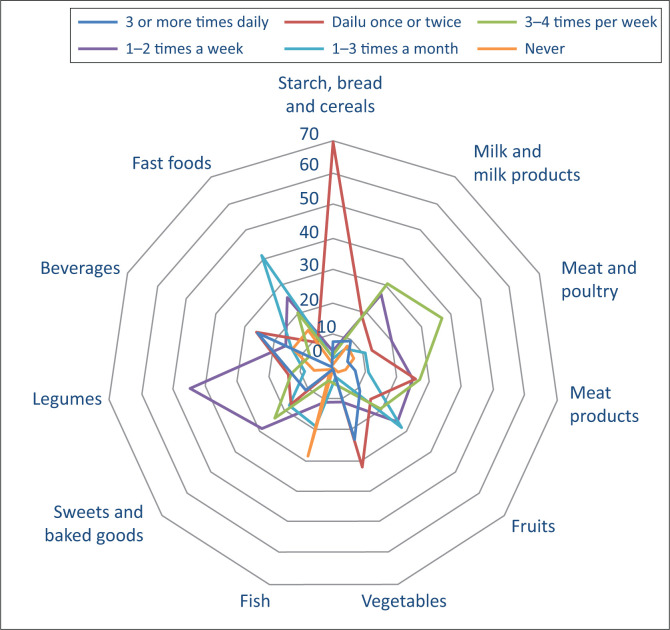
Frequency of consumption of dietary components.

Legumes, sweets and baked goods, beverages and fast foods demonstrated more sporadic intake patterns, with most respondents consuming these items monthly or weekly rather than daily. Notably, fast food showed one of the lowest consumption frequencies, with a sizable portion of respondents reporting consumption less than once a month or never. Fish consumption was the lowest among all food categories, with most individuals consuming fish only 1–2 times per month or less frequently (Figure 1).

The analysis identified three principal components with eigenvalues greater than 1, explaining a cumulative 62.3% variance in dietary intake. Specifically, Component 1 explained 30.5% of the variance, Component 2 explained 18.2% and Component 3 explained 13.6%. These components were considered to represent unique dietary patterns in our sample.

Within Component 1, high positive loadings were observed for staple foods (0.740), dairy (0.710), other fruits and vegetables (0.713), eggs (0.613), meat and poultry (0.720), sugar and sweets (0.702) and beverages (0.670). This pattern reflects a diet predominantly based on staple foods, animal products and sugary items. Individuals scoring high on this component likely consume a diet rich in these items, suggesting a traditional diet with a mix of staple grains, animal-based proteins and added sugars. The Component 2 pattern was characterised by high loadings on legumes (0.595) and snacks, fats, oils and condiments (0.452). Individuals with higher scores on this component tend to have a dietary pattern that includes a significant intake of legumes, snacks and other calorie-dense foods. This could indicate a diet influenced by processed and convenience foods supplemented with legumes, possibly reflecting a transitional or Westernised diet. The third component showed high loadings for vitamin-A-rich fruits and vegetables (0.578) and other fruits and vegetables (0.212), with negative loadings for fast foods (-0.490) and dairy (-0.305). This component suggests a dietary pattern emphasising fruits and vegetables, potentially healthier because of the inverse association with fast food consumption. Individuals with high scores on this component likely have a diet rich in fruits and vegetables, with limited intake of fast foods and dairy products (Table 3).

**TABLE 3:** Dietary patterns among adults in urban Zimbabwe.

Food group	Heavy starch	Legumes and snacks	High fruit and vegetable
Starch, bread, and cereals	**0.740**	0.089	0.101
Milk and milk products	**0.710**	0.067	−0.305
Meat and poultry	**0.720**	0.241	−0.027
Meat products	**0.613**	0.219	−0.214
Fruits	0.344	0.020	0.578
Vegetables	1.213	0.234	0.712
Fish	**0.558**	−0.485	0.200
Sweets and baked goods	0.382	0.552	−0.149
Legumes	0.272	**0.702**	0.150
Beverages	**0.670**	0.304	0.196
Fast foods	0.195	0.593	−0.490

Bold values highlight the highest value following Principal component analysis.

Individuals whose dietary consumption was classified as high fruit and vegetable had increased odds of being underweight. Additionally, the same individuals were 47% less likely to be classified as obese. However, there was no significant correlation between consuming a diet of legumes and snacks and either being underweight or obese (Table 4).

**TABLE 4:** Relationship between dietary pattern and double burden of malnutrition.

Variable	Underweight			Obese		
	AOR	95%CI	*p*	AOR	95%CI	*p*
**Dietary pattern**						
**Heavy starch**	R	-	-	-	-	-
**Legumes and snacks**	1.65	0.82–3.20	0.78	0.49	0.18–5.91	0.33
**High fruit and vegetable**	1.21	1.61–2.37	< 0.05	0.53	0.26–0.89	0.03

Note: Controlled for sex, age, location, socio-economic status, and employment.

AOR, Adjusted Odds Ratio; Cl, confidence interval.

## Discussion

This study reveals essential patterns in dietary intake, nutritional status and socio-economic influences on malnutrition. It aligns with prior studies while introducing perspectives on urban malnutrition in low- and middle-income countries (LMICs).

The study confirms the presence of a relationship between diet and both under and over-nutrition. While other patterns showed no association, consuming a diet high in vegetables was a risk for undernutrition, which could be attributed to a limited dietary diversity. Our findings support an earlier study conducted in Zimbabwe that found a significant relationship between diet and undernutrition among children.^25^ While we found individuals consuming a heavy starch dietary pattern to be insignificant in predicting either underweight or obesity, this is in contrast with a majority of studies where high-starch diets are often linked to obesity because of caloric density and frequent consumption patterns.^26,27^ While heavy starch may be the overall characteristic of the pattern, the relatively low caloric density and high satiety of traditional starch sources may help to explain this divergence and possible confounders such as physical activity.^28,29^

The socio-demographic analysis identified gender, age, household size and economic status as significant predictors of malnutrition. Men had higher odds of being underweight compared to women: a result consistent with studies suggesting that gender disparities in access to food and economic opportunities can affect men’s nutritional outcomes in urban LMIC settings.^30,31^ Our finding aligns with the global age-related trend, where older age groups tend to have higher obesity rates because of metabolic changes and reduced physical activity.^32^ The finding on household size and obesity could be explained by a possible association between family dynamics, economic resources, and food choices, where larger households may prioritise cheaper, energy-dense foods to accommodate family size. This finding is supported by evidence from other urban LMICs, where larger households often face economic pressures that lead to consuming calorie-dense, less nutritious foods.^33^

While providing valuable insights into the associations between dietary patterns, socioeconomic status and the double burden of malnutrition, this study has limitations inherent to its cross-sectional design. Cross-sectional studies cannot establish causality, meaning that the directionality and temporality of observed associations remain unclear. Additionally, the reliance on self-reported dietary data may introduce recall bias, potentially affecting the accuracy of dietary pattern assessments. Furthermore, cross-sectional studies face challenges in capturing the dynamic nature of dietary habits, influenced by factors such as the seasonal availability of certain food items, socioeconomic transitions, and their interplay over time. Despite these limitations, this study provides important baseline data on the relationship between dietary patterns, socioeconomic factors and the dual burden of undernutrition and overnutrition in the study population. These findings underscore the need for longitudinal research to understand causal pathways and temporal dynamics better.

## Conclusion

The patterns identified in this study underscore the need for targeted public health interventions to address the double burden of malnutrition in urban Zimbabwe. Policies promoting access to affordable, nutritious foods, particularly for vulnerable groups such as the youth and low-income families, could mitigate undernutrition and overnutrition. Nutrition education that emphasises the benefits of fruit and vegetable intake may also help shift dietary patterns away from energy-dense, nutrient-poor foods. Furthermore, as food inflation and economic instability continue to affect nutritional choices, government policies that stabilise food prices and support food security could alleviate some financial pressures contributing to malnutrition.

Further research should explore the long-term effects of these dietary patterns on health outcomes and the interplay between economic status and diet quality. Understanding how cultural preferences and food availability shape dietary patterns could enhance intervention strategies. This study contributes to our understanding of the double burden of malnutrition in urban Zimbabwe by illustrating the associations between dietary patterns, socio-demographic factors and malnutrition. Our findings highlight the critical role of economic constraints and food access in shaping malnutrition risks and suggest that promoting dietary diversity and food security is essential for effective malnutrition intervention. This adds an important picture for similar LMIC settings with a traditional focus on undernutrition within rural settings.

## References

[1] WHO. The double burden of malnutrition Policy brief [homepage on the Internet]. 2016 [cited 2024 Nov 02]. Available from: https://iris.who.int/bitstream/handle/10665/255413/WHO-NMH-?sequence=1

[2] WHO. Malnutrition key facts [homepage on the Internet]. 2024 [cited 2024 Nov 02]. Available from: https://www.who.int/news-room/fact-sheets/detail/malnutrition

[3] Fotso JC. Urban–rural differentials in child malnutrition: Trends and socioeconomic correlates in sub-Saharan Africa. Health Place. 2007;13(1):205–223. 10.1016/j.healthplace.2006.01.004 16563851

[4] Ali A, Sen S, Banerjee A, Chakma N. Rural-urban differentials in undernutrition among women in India: Evidence from a decomposition approach. Nutr Health. 2024;1-11:02601060241292401. 10.1177/02601060241292401 39469998

[5] Islam MS, Jhily NJ, Hasan R, et al. Nutritional transition in unindustrialized countries: Causes and consequences on public health. Indian J Public Health. 2021;12(4):343. 10.37506/ijphrd.v12i4.16567

[6] Batal M, Deaconu A, Steinhouse L. The nutrition transition and the double burden of malnutrition. In: Temple NJ, Wilson T, Jacobs DR, Bray GA, editors. Nutritional Health. Nutrition and Health [homepage on the Internet]. Cham: Springer International Publishing; 2023 [cited 2025 Jan 18]. p. 33–44. Available from: https://link.springer.com/10.1007/978-3-031-24663-0_3

[7] Jones AD, Acharya Y, Galway LP. Urbanicity gradients are associated with the household- and individual-level double burden of malnutrition in sub-Saharan Africa123. J Nutr. 2016;146(6):1257–1267. 10.3945/jn.115.226654 27170726

[8] Nnyepi MS, Gwisai N, Lekgoa M, Seru T. Evidence of nutrition transition in Southern Africa. Proc Nutr Soc. 2015;74(4):478–486. 10.1017/S0029665115000051 25686639

[9] Mbogori T, Kimmel K, Zhang M, Kandiah J, Wang Y. Nutrition transition and double burden of malnutrition in Africa: A case study of four selected countries with different social economic development. AIMS Public Health. 2020;7(3):425. 10.3934/publichealth.2020035 32968668 PMC7505783

[10] Steyn NP, Mchiza ZJ. Obesity and the nutrition transition in sub-Saharan Africa. Ann N Y Acad Sci. 2014;1311(1):88–101. 10.1111/nyas.12433 24725148

[11] Azzam A. Is the world converging to a ‘Western diet’? Public Health Nutr. 2021;24(2):309–317. 10.1017/S136898002000350X 33077024 PMC10195558

[12] Vorster HH, Kruger A, Margetts BM. The nutrition transition in Africa: Can it be steered into a more positive direction? Nutrients. 2011;3(4):429–441. 10.3390/nu3040429 22254104 PMC3257689

[13] Domingo A, Charles KA, Jacobs M, Brooker D, Hanning RM. Indigenous community perspectives of food security, sustainable food systems and strategies to enhance access to local and traditional healthy food for partnering Williams treaties first nations (Ontario, Canada). Int J Environ Res Public Health. 2021;18(9):4404. 10.3390/ijerph18094404 33919110 PMC8122547

[14] Carrera-Bastos P, Fontes O, Lindeberg S, Cordain L. The western diet and lifestyle and diseases of civilization. Res Rep Clin Cardiol. 2011;2011:15–35. 10.2147/RRCC.S16919

[15] Abubakari MM, Anaman KA, Ahene-Codjoe AA. Urbanization and arable land use in northern Ghana: A case study of the Sagnarigu municipality in the greater tamale area. Appl Econ Finance. 2022;9(1):68–84. 10.11114/aef.v9i1.5469

[16] Casari S, Di Paola M, Banci E, et al. Changing dietary habits: The impact of urbanization and rising socio-economic status in families from Burkina Faso in sub-Saharan Africa. Nutrients. 2022;14(9):1782. 10.3390/nu14091782 35565752 PMC9104313

[17] Smart JC, Tschirley D, Smart F. Diet quality and urbanization in Mozambique. Food Nutr Bull. 2020;41(3):298–317. 10.1177/0379572120930123 32700560

[18] Vilar-Compte M, Burrola-Méndez S, Lozano-Marrufo A, et al. Urban poverty and nutrition challenges associated with accessibility to a healthy diet: A global systematic literature review. Int J Equity Health. 2021;20(1):40. 10.1186/s12939-020-01330-0 33472636 PMC7816472

[19] Guevara-Romero E, Flórez-García V, Egede LE, Yan A. Factors associated with the double burden of malnutrition at the household level: A scoping review. Crit Rev Food Sci Nutr. 2022;62(25):6961–6972. 10.1080/10408398.2021.1908954 33840313

[20] WHO. A healthy lifestyle – WHO recommendations [homepage on the Internet]. 2010 [cited 2025 Jan 17]. Available from: https://www.who.int/europe/news-room/fact-sheets/item/a-healthy-lifestyle---who-recommendations

[21] Rutstein SO. Steps to constructing the new DHS Wealth Index. Rockv MD ICF Int [homepage on the Internet]. 2015 [cited 2025 Jan 17];6. Available from: https://preview.dhsprogram.com/programming/wealth%20index/Steps_to_constructing_the_new_DHS_Wealth_Index.pdf

[22] Howe LD. The wealth index as a measure of socio-economic position [homepage on the Internet]. PhD Thesis. London School of Hygiene & Tropical Medicine; 2009 [cited 2025 Jan 17]. Available from: https://researchonline.lshtm.ac.uk/id/eprint/768490

[23] FAO. A resource guide to method selection and application in low resource settings [homepage on the Internet]. Rome: FAO; 2018 [cited 2025 Jan 17], p. 152. Available from: https://openknowledge.fao.org/server/api/core/bitstreams/3dc75cfc-9128-4f29-9d76-8d1f792078f0/content

[24] Food and Nutrition Committee. Zimbabwe livelihoods assessments report, 2024 [homepage on the Internet]. 2024 [cited 2025 Jan 17]. Available from: https://www.unicef.org/zimbabwe/reports/zimbabwe-livelihoods-assessment-report-2024

[25] Marume A, Archary M, Mahomed S. Dietary patterns and childhood stunting in Zimbabwe. BMC Nutr. 2022;8(1):111. 10.1186/s40795-022-00607-7 36224638 PMC9555084

[26] Jiang K, Zhang Z, Fullington LA, et al. Dietary patterns and obesity in Chinese adults: A systematic review and meta-analysis. Nutrients. 2022;14(22):4911. 10.3390/nu14224911 36432596 PMC9698822

[27] Seifu CN, Fahey PP, Hailemariam TG, Frost SA, Atlantis E. Dietary patterns associated with obesity outcomes in adults: An umbrella review of systematic reviews. Public Health Nutr. 2021;24(18):6390–6414. 10.1017/S1368980021000823 33612135 PMC11148623

[28] Maulina YR, Margawati A, Purwanti R, Tsani AFA. Differences in eating habits and physical activity before and during distance learning. J Gizi Indones Indones J Nutr. 2022;10(2):122–134. 10.14710/jgi.10.2.122-134

[29] Kayode OO, Alabi QK. Food consumption patterns, physical activity and overweight and obesity among undergraduates of a private university in Nigeria. Clin Nutr Exp. 2020;31:28–34. 10.1016/j.yclnex.2020.01.001

[30] Abate T, Mengistu B, Atnafu A, Derso T. Malnutrition and its determinants among older adults people in Addis Ababa, Ethiopia. BMC Geriatr. 2020;20(1):498. 10.1186/s12877-020-01917-w 33228557 PMC7684921

[31] Little M, Humphries S, Dodd W, Patel K, Dewey C. Socio-demographic patterning of the individual-level double burden of malnutrition in a rural population in South India: A cross-sectional study. BMC Public Health. 2020;20(1):675. 10.1186/s12889-020-08679-5 32404080 PMC7218837

[32] Bardon LA, Corish CA, Lane M, et al. Ageing rate of older adults affects the factors associated with, and the determinants of malnutrition in the community: A systematic review and narrative synthesis. BMC Geriatr. 2021;21(1):676. 10.1186/s12877-021-02583-2 34863118 PMC8642873

[33] Cediel G, Perez E, Gaitán D, Sarmiento OL, Gonzalez L. Association of all forms of malnutrition and socioeconomic status, educational level and ethnicity in Colombian children and non-pregnant women. Public Health Nutr. 2020;23(S1):s51–s58. 10.1017/S1368980019004257 32131920 PMC10200449

